# Normative study of 500 general-knowledge of true-false questions for Russian young adults

**DOI:** 10.1371/journal.pone.0300600

**Published:** 2024-04-29

**Authors:** Beatriz Martín-Luengo, Oksana Zinchenko, Aleksandra Dolgoarshinnaia, Maria Alekseeva

**Affiliations:** Institute for Cognitive Neuroscience, Centre for Cognition and Decision Making, HSE University, Moscow, Russian Federation; Tallinn University: Tallinna Ulikool, ESTONIA

## Abstract

The main aim of this study was to validate 500 true-false general-knowledge questions in Russian. These norms are valuable to researchers in many fields, as is shown by the impact and relevance of similar norms available in other languages. Although the Russian language is widely spoken, there are no norms available in this language for this type of questions. True-false questions are very useful for measuring semantic memory, among other topics, in neurocognitive studies where there is a trade-off between experimental time and the need for many trials. These types of experimental materials are heavily rooted in cultural background knowledge, making the mere translation from one language to another insufficient. The present research aims to fill this gap. One hundred fifty-five participants answered 500 true-false general knowledge questions split over several consecutive days and three topics: Social Sciences, Natural Sciences, and Culture & Sport. The participants’ task was to indicate whether the statements were true or not, as well as the confidence they had in the correctness of their answer. Despite obtaining questions on each of the topics covering all difficulty levels, grouped analyses showed that Social Science’s accuracy was higher than for Natural Science’s or Culture & Sport questions. In relation to confidence, the grouped perceived difficulty was higher for questions about Culture & Sports when compared with the other two topics. Thus, this study reports and makes available a large pool of Russian true-false general knowledge questions covering different levels of difficulty.

## Introduction

General knowledge questions are a valuable experimental stimulus that can be used to measure semantic memory processes [1,2]. General knowledge questions can also be used in other multiple strands of research such as tip-of-the-tongue studies [3], errors in adults [4], errors in children [5], and false memory research [6]. These type of valuable stimuli requires of normative studies to assure the cultural alignment of the content of the questions with the target sample since the mere translation from one language to another is insufficient [7–11]. Databases also need to be updated from time to time to adjust the questions to continuing cultural changes [11], and to the different capacities of the population as for example the cognitive decline due to age [12]. This type of question have also been the focus of research about the testing-effect for which researchers tried to find the most optimal tool to aid the retrieval of information. For example, in a series of four experiments [13] it was compared the long-term retention of text passages. The participants’ tasks were either answering true-false questions, short-answer questions, or restudying the texts. True-false questions yield better retrieval performance compared to the other two learning strategies in isolation. Uner’s study [13] also expands the need to better understand how this particular type of question cognitively operates and consequently, the need for normative studies to further explore it. In this study we aimed to create the first database of general knowledge questions in a true-false format in the Russian language.

There are different formats for closed questions [14,15]. For example, in multiple choice questions the participant is presented with one question and several alternatives and need to choose which of those alternatives is the correct one (for other formats of multiple-choice questions see [16]). In the two-alternative forced-choice format participants are presented with a question and alternatives as with the multiple-choice format, but in this case the number of alternatives is only two. Finally, in true-false questions two alternatives are provided: true, false. That is, participants’ task is to decide whether the statement presented is true or not.

The format of the questions influences memory and metamemory performance. True-false questions have been found to be more difficult and result in worse performance when compared with two alternative forced-choice (2AFC) tests [17]. In a true-false test participants have less information at hand to decide which of the alternatives is the correct answer. For example, if someone has no clue about what the capital of Malawi is, their choice between true and false for this sentence: ¨Lilongwe is the capital city of Malawi” will be random. However, if as option for the question: “What is the capital of Malawi?” are presented “Lilingwe” and “Pretoria”, someone might discard Pretoria knowing that is one of the three capital cities of South Africa. That is, the alternatives presented in a 2AFC test might aid the decision over which alternative to choose because they provide additional information.

However, there are also some limitations and precautions that need to be considered when selecting this type of question. For example, in true-false questions, as well as 2AFC, respondents have a 50% chance of guessing which impacts the reliability of these types of questions [18]. If the questions are not carefully written, it might be easier for respondents to detect flaws that would indicate that the correct answer is false [19,20]. Finally, when these types of questions are used to test the knowledge of a course, this format might lead students to memorize, rather than understand, the content [21].

The type of memory test also influences the metamemory performance. Several studies have shown that confidence judgments allow the participants to make finer grained distinctions between answers [22], even improving metacognitive monitoring [23]. For example, previous studies have reported that retrospective confidence is a better predictor of accuracy when it is associated with multiple-choice questions than with other types of questions [24,25]. Other studies also found that by comparing metamemory performance from multiple-choice questions with two-alternative and true-false questions, participants adopted a more conservative criterion of answering for the true-false questions, presumably due to the lower access of information that this type of question format provides [26].

True-false questions are widely used to assess student learning [14,27]. As a tool in research they have been used to study the boundaries of the calibration of subjective probability [28], the effect of feedback [26], and to test its retention benefits for its use as a learning tool [13]. One of the main advantages of this format is that participants require less time to answer the questions and this is an asset in experiments that require many trials. These types of questions measure participants’ knowledge in a more direct way because there are no alternatives that participants can use as a cue (i.e., choosing by discarding other options). In sum, the evidence points towards objective and subjective differences in the questions in true-false format when compared with other closed format questions. These types of questions are very useful in time limited experiments and at the same time they provide a less biased measure of participants’ knowledge. The literature review shows that the characteristics from one format of closed questions to another might dramatically change. Specific normative studies need to be run in order to gather reliable indices for these questions.

The main goal of the present work is to create a large normative database of general knowledge questions in a true-false format oriented to the young Russian population. To this aim, questions covering different topics and with a range of difficulties were selected and presented to a representative sample of participants. As done in similar studies [8,9], accuracy and the subjective experience of correctness—confidence—was computed for each question and the general features of the questions described.

## Methods

### Participants

The data collection of this study started in November 2022 and ended in February 2023. The data was anonymized. We set the following requirements to be invited to take part in our online study: to lie within the age range of 20–35 years old and to have completed, at least, basic elementary school. We selected this age range because this is the target age on the majority of the cognitive studies and this is the first study of this kind in Russian. One hundred forty-five participants, ninety of them females (general *M* age = 23.3, *SD* = 5.2; for males *M* age = 24.2, *SD* = 6.0 for female *M* age = 22.8, *SD* = 4.6) were recruited via social media (Facebook, Vkontakte, Telegram) and answered online questionnaires. This sample is similar to the one used in normative studies where all participants provide responses to all items (N = 103 participants in [9]). Their participation was voluntary without any monetary or other compensation. Participants reported a similar educational level. Among the 145 participants 67 (46%; 41 females, 26 males) reported a high educational level (bachelor’s or master’s degree finished, or were PhD students), 13 (~9%; 9 females and 4 male) were bachelor students, and 65 (~45%; 40 females, 25 male) had finished high school.

### Materials

This study was reviewed and approved by the HSE University Ethics Committee. The participants provided their written informed consent to participate in this study. Five hundred general knowledge questions (GKQ) were used in this experiment. The questions covered different topics from Natural Sciences (259 questions), Social Sciences (104), and Culture & Sports (137). Three hundred five of the questions used for creating this database are transformations from other normative studies [9]. In the Supplemental materials (https://osf.io/pe6d3/?view_only=f7ef2eb26dae401f99f8f381bff7d0f5) we have included a column indicating whether the question was new or transformed. Those new were retrieved from two quizzes websites, https://iq2u.ru and https://baza-otvetov.ru, the first offering examples of the questions used in the Unified State Exam (a compulsory standardized examination taken in order to receive a high school diploma), the second being oriented at a more general audience. The final set included 500 unique questions (if there were two questions asking for similar information, we considered only one of them) with four-alternative answer options from general knowledge categories provided by the websites. The questions were later sorted as belonging to one of the three main topics—Natural sciences, Social sciences, and Culture & Sports. The format of the questions was subsequently changed into a true or false statement by choosing either the correct answer or one of the incorrect alternatives. The difficulty of the questions was determined in two ways: questions that required specific knowledge of a topic, as for example terminology, historical dates, and names of historical figures; questions that contained the incorrect alternative that resembled the correct answer in terms of plausibility. An example for this latter case: “Mermaid is the symbol of Warsaw” is a correct statement with high level of difficulty as mermaid is mostly associated with Copenhagen because of the statue at the Langelinie promenade; “A triangle with sides of 3, 4, and 5 meters is called the Greek triangle” an incorrect statement with high level of difficulty as it contains specific terminology. After that the difficulty was once again independently evaluated by two university degree holders, both Russian native speakers. If the evaluators did not agree on the level of difficulty, the question was substituted with a new one until the proportion of easy and difficult correct and incorrect answers was achieved (overall, 16 questions were substituted in such a manner). For half of the questions the correct answer was “True” and for the other half “False”.

### Procedure

The experiment was conducted using google forms. The questions were divided into five tests and delivered to participants over a period of five days (1 day—1 test). For each statement, participants needed to indicate whether it was true or false and provide their confidence in the correctness of their choice. In particular, participants’ answers were collected using the drop-down answer option with the following alternatives: “true—completely sure”, “true—highly sure”, “true—moderately sure”, “true—not sure”, “true—totally unsure”, “false—completely sure”, “false—highly sure”, “false—moderately sure”, “false—not sure”, “false—totally unsure”. Confidence evaluations were coded as follows: completely sure– 100%, highly sure– 80%, moderately sure– 50%, not sure– 30%, totally unsure– 0%. After completing each of the tests, participants were provided with a general feedback over their performance of that test. The order of the five tests and the order of the questions within each test was randomized test.

## Results and discussion

There were twelve questions always correctly answered, nine of them the correct answer was True. None of the questions were consistently answered incorrectly. For the list of questions used, their accuracy and confidence ratings measured collectively and split by sex, see the Supplementary material file.

### Accuracy

**Fig 1:**
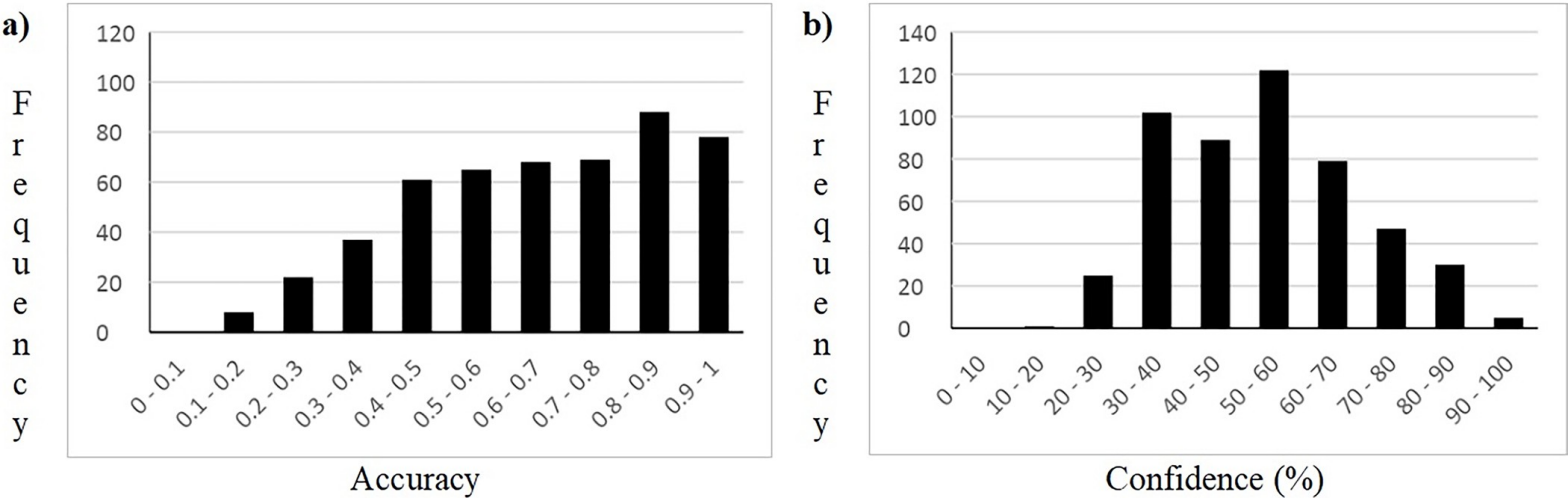
Accuracy and confidence frequency distribution of answers for all questions.

Compared to other types of questions, such as cued recall and free recall, recognition questions are mainly based on familiarity [29–31], and therefore it is more difficult to create questions covering all levels of difficulty [9]. See Fig 1A for the distribution of the answer accuracy for all questions. The sample of questions tested covered all ranges of difficulty being a bit skewed towards easier questions. None of the questions were incorrectly answered by all participants. However, the lack of questions that were consistently wrongly answered by all participants is not necessarily a negative output because most of the time these types of questions correspond to the so-called deceptive or misleading questions [32,33]. For example, Madrid is the capital city of Spain, but it is not unusual to find people that believe it is Barcelona based on their familiarity due to the widely international projection. Another very similar example is with the capital of Australia, many people will answer highly confidently that it is Sydney instead of Canberra.

**Fig 2:**
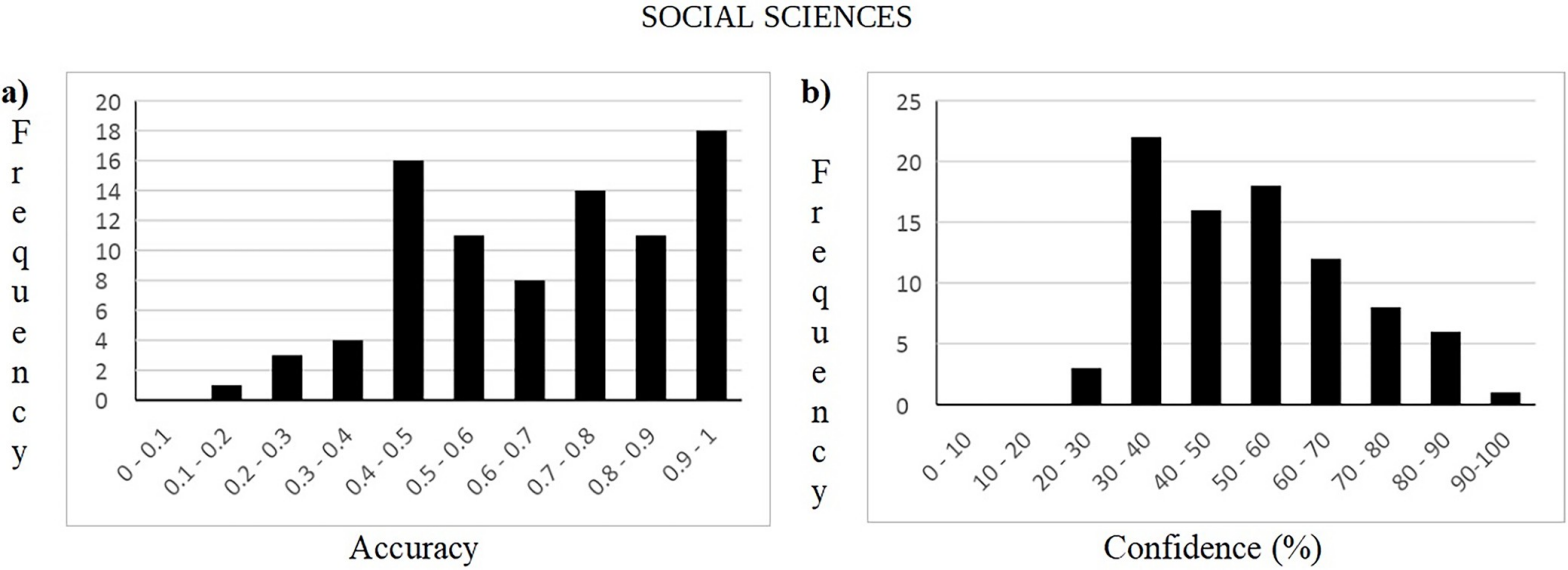
Accuracy and confidence frequency distribution of answers for social science questions.

**Fig 3:**
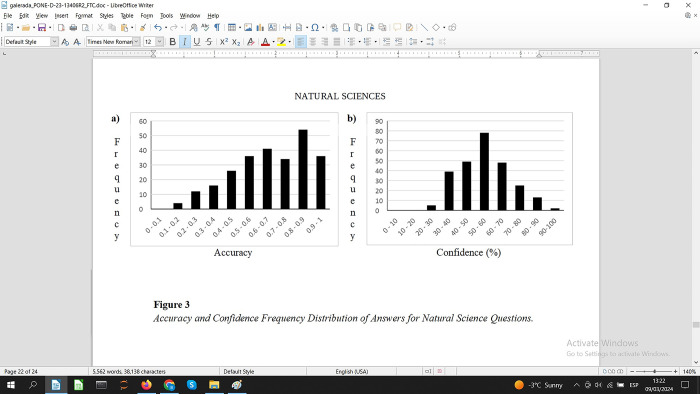
Accuracy and confidence frequency distribution of answers for natural science questions.

**Fig 4:**
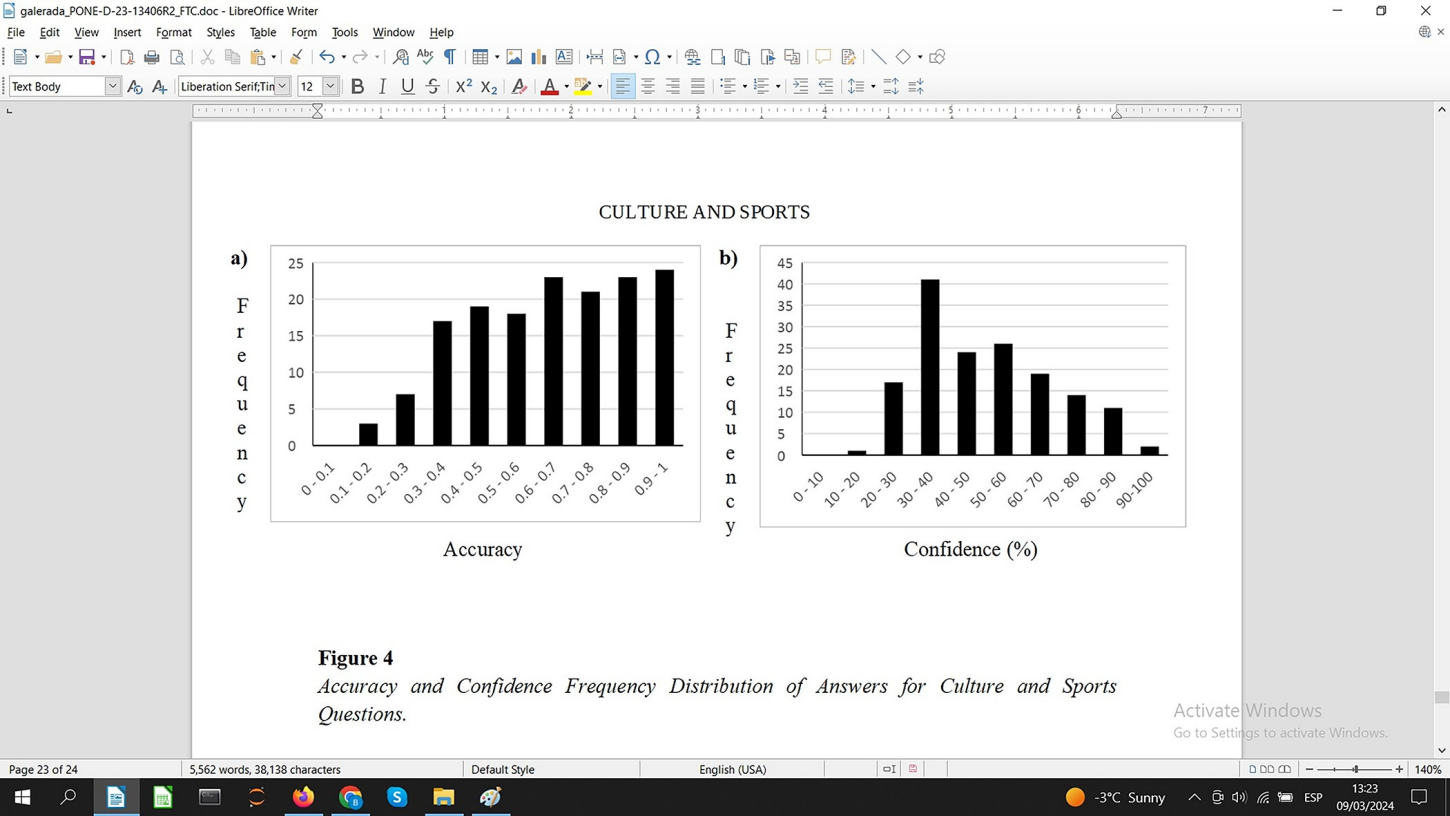
Accuracy and confidence frequency distribution of answers for culture and sports questions.

An inspection by topic, see the A panels in Figs 2–4, shows that the Culture & Sport questions have a more balanced number of questions for each degree of difficulty. Social and Natural Sciences questions also have a number of questions on each level of difficulty although the distributions are a bit more unbalanced.

To compare differences between categories not taking sex into account, we first performed single-factor ANOVA on Natural Sciences, Culture & Sports, and Social Sciences on accuracy. The results showed significant differences in accuracy scores between the three categories (*F*(2, 434) = 32.30, *p* < .001). To investigate these differences, we performed independent Student’s t-tests, applying Bonferroni correction. Accuracy on Social Sciences was significantly higher than on Natural Sciences (.69 vs .67; *t*(288) = 2.52, *p* = .012, *d* = .296), while Accuracy on Social Sciences was also significantly higher than on Culture & Sports (.69 vs .63; *t*(288) = 7.93; *p* < .001, *d* = .931) and significantly higher on Natural Sciences than on Culture & Sports (.67 vs .63; *t*(288) = 5.27, *p* < .001, *d* = .619).

We did not observe any differences in accuracy between male and female participants for all questions or for the subcategories (see Table 1). These results show that our questions do not have sex-related biases and can be used for the general population of Russian young adults.

**Table 1:** Mean accuracy (SD) split by sex (N female = 90; N male = 55) and topic with statistical analysis of between-gender differences.

Topic	Sex	Mean accuracy (*SD*)	Student’s *t* test	Cohen’s *d*
All questions	Female	.66 (.06)	*t*(143) = .590, *p* = .556	.101
	Male	.67 (.07)		
Social sciences	Female	.67 (.06)	*t*(143) = 1.083, *p* = .281	.185
	Male	.68 (.08)		
Natural sciences	Female	.67 (.07)	*t*(143) = 1.107, *p* = .270	.189
	Male	.68 (.07)		
Culture and sports	Female	.65 (.07)	*t*(143) = -.796, *p* = .428	-.073
	Male	.64 (.07)		

Bonferroni correction for multiple comparisons set the significance at p = 0.01.

### Confidence

See Fig 1B for the general distribution of confidence among all questions. Subjective confidence ratings in questions where the option of the answers are provided are particularly relevant since the accuracy ratings might be distorted with participants’ chance performance by selecting one option. However, subjective confidence ratings provide information about the perceived difficulty and can help to detect deceptive questions, i.e. those incorrectly answered but assessed with a high-confidence rating [34].

In the examination of the frequency chart for accuracy for all questions it is noticeable that although there is a similar proportion of answers with an accuracy between .5 to .8, and slightly higher for .8 to .9, most of the confidence ratings are concentrated between 30 and 60%. That is, the subjective perception of participants is that the questions were more difficult than they actually were.

Participants’ subjective perception about the difficulty of the questions varies by topic, see Figs 2–4, panel B. Based on the peaks for each of the frequency charts, the questions for Social Sciences and Culture & Sports were considered more difficult compared to Natural Science questions. Confidence ratings for Social Sciences and Culture & Sports are 30–40% whereas for Natural Sciences questions the peak is 50–60%. Despite this small numerical difference in confidence, these results indicate a balanced perception of difficulty regardless of the topic of the questions.

To compare differences between categories not taking sex into account, we performed single-factor ANOVA on Natural Sciences, Culture & Sports, and Social Sciences on the confidence ratings. The results suggest there are significant differences in the confidence ratings between the three categories (*F*(2, 434) = 23.59, *p* < .001). To further investigate these differences, we performed independent Student’s t-tests applying Bonferroni correction. Confidence ratings on Social Sciences were significantly higher than on Culture & Sports (56.41 vs 46.97; *t*(288) = 6.24, *p* < .001, *d* = .733), while there was no significant difference in confidence between Social Sciences and Natural Sciences (56.41 vs 55.05; *t*(288) = .94, *p* = .35, *d* = .110). When we compared confidence on Culture & Sports and Natural Sciences, we found that confidence on Natural Sciences was significantly higher (55.05 vs 46.97; *t*(288) = 5.43; *p* < .001, *d* = .638).

Finally, we did not observe any differences in confidence ratings between male and female participants for all questions or for the subcategories (see Table 2). These results suggest that in general the perceived difficulty of our questions was not related to sex-related biases and can, therefore, be used for the population of young Russian adults.

**Table 2:** Mean confidence (SD) split by sex (N female = 90; N male = 55) and topic with statistical analysis of between-gender differences.

Topic	Sex	Mean confidence (SD)	Student’s *t* test	Cohen’s *d*
All questions	Female	52.52 (10.94)	*t*(143) = .783, *p* = .435	.134
	Male	54.10 (13.21)		
Social sciences	Female	51.82 (12.0)	*t*(143) = 1.209, *p* = .229	.207
	Male	54.53 (14.78)		
Natural sciences	Female	54.06 (11.46)	*t*(143) = 1.251, *p* = .213	.214
	Male	56.66 (13.26)		
Culture and sports	Female	50.33 (11.92)	*t*(143) = -.339, *p* = .735	-.058
	Male	49.59 (13.85)		

Bonferroni correction for multiple comparisons set the significance at p = 0.01.

### Calibration

**Fig 5:**
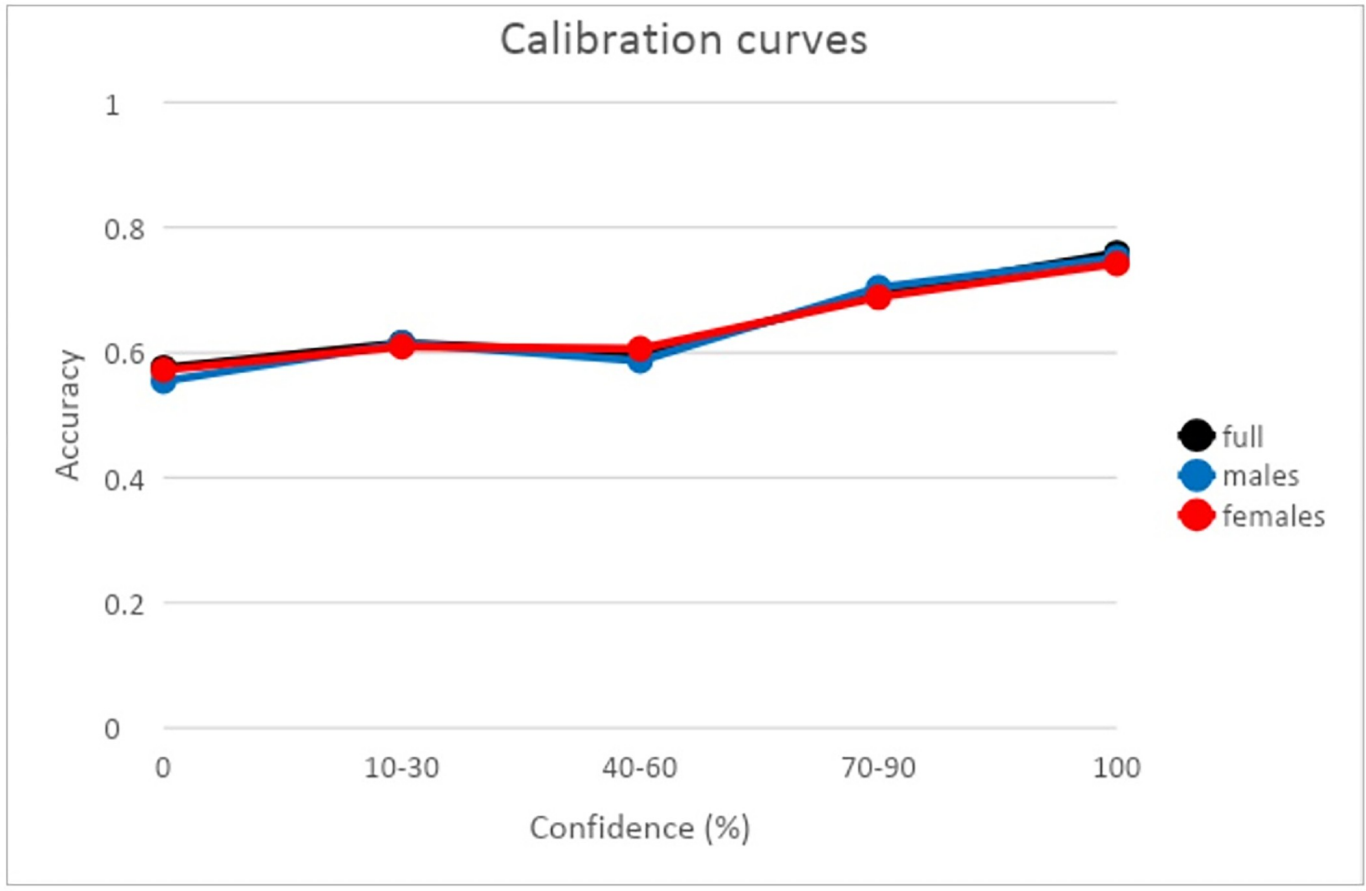
Calibration curves, for full sample, female and male separately. O% and 100% confidence answers were not merge with any other confidence rating because of their qualitative differences in terms of deliberative process involved to answer them (Luna et al., 2015) [6].

The graphical representation of the confidence-accuracy calibration curves is remarkably useful to study the correspondence of both measures. In a calibration chart, the x-axis usually represents the levels of confidence (subjective measure) and the y-axis represents the levels of accuracy (objective measure) [35]. A perfect calibration curve is represented by the diagonal where equal levels of accuracy and confidence converge. In this case, participants show that they have a perfect subjective estimation of their accuracy. When the curve lies below the diagonal, in this case participants show overconfidence because a bigger confidence rating is attributed to a lower accuracy performance. If the curve is above the diagonal this indicates that participants are under-confident because their ratings of confidence are lower in relation to their accuracy. See Fig 5 for calibration curves for all samples as well as for only females and only males. The three calibration curves do not differ at any confidence level and present a large overlap. The calibration curves also present a rather marked “hard-easy” effect [6,9,36]. The “hard-easy” effect exemplifies in easy tasks an underestimation of our abilities but an overestimation in difficult tasks. In this particular case it denotes that participants rated easy questions with low confidence, that is, questions with a high/moderate accuracy, and that they selected higher confidence for difficult questions. This is a normal result often found in calibration curves regardless of the memory test used.

## Conclusions

This study reports norms for general-knowledge questions in a true-false format in Russian. It is important to note that we used a traditional true-false format for the answers with a twist in which participants, apart from selecting true or false, also rate their answers for confidence within five levels for true and another five levels for false. This modification allowed us to collect more information about the difficulty perceived by participants, therefore, more information about the quality of the questions. Finally, seeing that there were no significant differences in accuracy and confidence between female and male participants in general and related to the specific the sub-categories of questions, we can argue that our results can be generalized for the young Russian population.

In summary, this report provides a new tool for researchers including information of the accuracy and also the participants’ subjective confidence in their answers.

### Limitations

Our study has some limitations as, for example, the participants’ age range. Previous studies have pointed to the need for norms adequate to the cognitive capacities of participants [12]. Considering that our sample is young, these norms should be carefully applied to other populations such as older people who may have some level of cognitive decline. Another limitation is related to the set of questions from the topic Culture & Sports. This particular set of questions will also require careful consideration from researchers before being used since they more quickly become outdated. Therefore, the difficulty ranking—objective and subjective—might change for Culture & Sport questions from the one reported here.

This type of normative research is needed and valuable to develop experiments in different cognitive fields [1–6,13]. However, it is also equally important that in particular questions on culture and sport are updated from time to time [11].

Finally, some readers may express concern about the validity of our results considering the online nature of our study, therefore, possible limitations need to be addressed. In recent years there has been a noticeable trend to move behavioral studies to online formats and platforms. Consequently, many questions were raised as to the validity of the results of such studies. Some points of criticism include sampling biases, inattentiveness of participants and possible cheating behaviors (for review see [37]). However, most of these concerns were discussed in relation to online-platforms such as MTurk, Qualtrics, Prolific Academic and others, where participants take part in experiments and surveys “professionally” [38–40]. Our participants were recruited through different social media platforms and advertisements, communicated with the experimenters directly which ensured that they were real people (and not bots) and did not participate in the study multiple times. The design of the study also allowed us to keep participants alert and attentive. First, the entire poll of statements was divided into subsets solved over several days, so that participants were not overwhelmed and exhausted by the number of questions. Second, the answer that they were supposed to give required assessment of their subjective feelings about the question which also prompted them to pay attention. As for cheating behavior, participants with the highest accuracy only reached an accuracy of .83 (95% CI [.80,.86]), which seems low for cheating.

Finally, many studies, including normative ones, found no significant differences between the data obtained online and in-person that would compromise the data as invalid or untrustworthy [12,41–45]. It can be concluded, therefore, that well-controlled online studies allow data to be obtained from a more diverse sample compared to many laboratory studies that, for instance, recruit university students in exchange for course credit [37].

We could not control for some factors due to the online format. First, despite the clear instructions to find a quiet place to run the experiment, we could not guarantee that there were no sudden distractions. Second, we could not visually assess participants’ mood. In a laboratory setting researchers might notice that participants arrive tired or in a bad mood and can recommend postponing the experiment for some time or rescheduling to another day. In fairness, the opposite situations can also occur as online studies give participants flexibility to perform the task when they feel well-rested. Taking this into account, it would be a good idea both for online and in-person studies to include assessments of participants’ mood before starting the experimental tasks.

As a final point we would like to draw the attention to the different types of information that is collected and analyzed in this type of normative studies. In our case, we focused on accuracy, subjective confidence, and sex. In some other studies, for example in a recent study [12], reaction times were also collected. In our particular case, being an online study, this measure could have not been reliable since it will depend on the internet speed and computer used, but certainly a measure to be included in laboratory experiments. In another study [1], errors were analyzed. Because of the type of answer in a true-false format it would be redundant to run this analysis, yet, in other formats such as in multiple choice questions, it is a helpful measure to be included [9]. Thus, we recommend including more measures that are feasible to collect in this type of study, increasing the information about the quality of the normed questions.
